# Cutting Dipping Application of Flupyradifurone against Cassava Whiteflies *Bemisia tabaci* and Impact on Its Parasitism in Cassava

**DOI:** 10.3390/insects14100796

**Published:** 2023-09-30

**Authors:** Ruben Caspary, Everlyne N. Wosula, Khamis A. Issa, Massoud Amour, James P. Legg

**Affiliations:** 1 Faculty of Natural Sciences, Department Biology, Friedrich-Alexander-Universität Erlangen-Nürnberg (FAU), Steinknöck 11, 91054 Erlangen, Germany; ruben.caspary@fau.de; 2 International Institute of Tropical Agriculture, Dar es Salaam P.O. Box 34441, Tanzania; k.issa@cgiar.org (K.A.I.); a.massoud@cgiar.org (M.A.); j.legg@cgiar.org (J.P.L.)

**Keywords:** flupyradifurone, parasitoids, *Encarsia* spp., *Eretmocerus* spp., CMD, CBSD, IPM

## Abstract

**Simple Summary:**

The results from this study, conducted under farmer field conditions, show that application of the insecticide flupyradifurone through cassava cutting dips is effective at reducing whitefly numbers and cassava mosaic disease incidence and increasing cassava root yield. Insecticide application through cutting dips is geared as an integrated pest management strategy that could limit the trophic transfer of pesticides. We found that the rate of parasitism by individual parasitoid species was not significantly affected by the application of flupyradifurone. This strategy could be scaled further for the management of whiteflies in cassava, while at the same time minimizing the impact on non-target beneficial insects.

**Abstract:**

The cassava whitefly *Bemisia tabaci* causes damage in cassava through the feeding and vectoring of plant viruses that cause cassava mosaic and cassava brown streak diseases. This study sought to explore the efficacy of cutting dipping in flupyradifurone for whitefly control and the impact of the mode of application on whitefly parasitism under farmer field conditions. The insecticide treatment significantly reduced adult whiteflies by 41%, nymphs by 64%, and cassava mosaic disease (CMD) incidence by 16% and increased root yield by 49%. The whitefly parasitism rate by *Encarsia* spp. parasitoids was 27.3 and 21.1%, while *Eretmocerus* spp. had 26.7 and 18.0% in control and flupyradifurone, respectively, and these differences were not significant. Electropenetrography recordings of whitefly feeding behaviour on flupyradifurone-treated plants showed significantly reduced probing activity and a delay in reaching the phloem as compared to the control. The findings from this study demonstrated that cassava cutting dipping in flupyradifurone significantly reduces whitefly numbers and cassava mosaic disease incidence, thus contributing to a significant root yield increase in cassava. Flupyradifurone applied through cutting dips does not significantly impact parasitism rates in cassava fields. Routine monitoring of parasitoids and predators in insecticide-treated versus control fields should be emphasized to determine the impact of pesticides on these beneficial non-target organisms.

## 1. Introduction

The whitefly, *Bemisia tabaci* (Gennadius) (Aleyrodidae, Hemiptera), is one of the most economically damaging pest species of crop plants in Africa [1,2]. It causes physical damage as well as transmits many plant viruses. Besides the importance on cassava, the species complex is well-known across the world as one of the most important global pests and has been reported on 500 plant species in 74 families [3]. Severe prolonged whitefly infestations coupled with sooty mould, leaf chlorosis, and leaf fall results in considerable root yield reduction in cassava [4]. In sub-Saharan Africa, *B. tabaci* is considered a major pest of cassava because it vectors viruses that cause two major diseases that negatively impact agronomic and economic aspects of cassava production. These are cassava mosaic disease (CMD) and cassava brown streak disease (CBSD) [5]. Overall, these viruses affect more than half of all cassava plants in sub-Saharan Africa and cause annual losses of more than USD 1 billion [6].

Insecticide development against *B. tabaci* was undertaken following outbreaks across North America in the 1990s. The compounds used were conventional neurotoxic compounds, such as bifenthin, insect growth regulators (IGRs), and most recently neonicotinoids. Reports show that pyrethroids were most efficacious, however, as spray mixtures, as the compounds used alone only provided minimal protection against *B. tabaci* [7]. Nicotinic accetylcholine receptor agonists (nAChR) present the newest groups of insecticides, with a unique systemic mode of action due to high water solubility and long residual efficacy. Among nAChR agonists, there are different classes, such as neonicotinoids and butenolides. Metabolized neonicotinoids are found to have a tenfold greater toxicity than the initial compound. Neonicotinoids can be applied as foliar sprays but also with other methods, such as soil drenches, subsurface granule application, and seed treatments. Imidacloprid was the first neonicotinoid that was widely used for the management of *B. tabaci* on vegetable crops [7].

*B. tabaci* is a cryptic species complex with the cassava-colonizing type identified based on mitochondrial DNA cytochrome oxidase I (COI) sequencing as sub-Saharan Africa (SSA), which has five groups (SSA1–5), with the major one, SSA1, divided into subgroups (SSA1-SG1 to SG5) [8,9]. Recently, six major haplogroups of cassava *B. tabaci* were defined (SSA-ECA, SSA-ESA, SSA-CA, SSA-WA, SSA2, SSA4) based on more robust SNP genotyping [10]. *B. tabaci* cryptic species respond differently to insecticides, with some, such as MEAM1 and MED, developing resistance to a wide range of current chemistries [11]. The cryptic species that is predominant in the study area is the haplogroup SSA-ESA (mitotype SSA1-SG3) [9,10].

Flupyradifurone, registered name Sivanto (SL 200), is a systemic insecticide with flexible modes of application and is mainly intended for the control of sucking pests, such as aphids, hoppers, and whiteflies. Flupyradifurone acts as a nicotinic acetylcholine receptor agonist, such as the neonicotinoids; however, it is included in the class of butenolide insecticides based on its butenolide scaffold. It acts on a variety of pests with piercing and sucking mouthparts with high selectivity, quick action, and long-lasting effects [12]. Flupyradifurone has particular promise as an insecticide due to its relatively safe toxicological and ecotoxicological profile and versatility in application [12]. It is reported to be effective on nicotinoid-resistant *B. tabaci* in cotton, opening new options for resistance management. Flupyradifurone has been evaluated using various assays under laboratory and field conditions and is reported to be among the most effective insecticides against adult *B. tabaci* whiteflies [13,14,15]. Flupyradifurone has also been shown to control whitefly-transmitted viruses through the suppression of transmission [16,17]. Promising advantages over imidacloprid, thiamethoxam, and clothianidin were highlighted by findings of a 100-fold lower toxicity to honeybees, also considering the sublethal effects and low acute contact toxicity [18].

Parasitoids have been recognized as effective biocontrol agents against a broad range of agricultural pests, and many have been commercially developed for biocontrol for several crops [19]. Parasitoids have several benefits, especially in the IPM context. Parasitoids have been shown to be permanent pest managers outside of target fields, as they provide similar levels of *B. tabaci* parasitism on other herbaceous plants surrounding cassava fields [20]. Not only are natural enemies sustainable and safe, but they are inexpensive, once naturalized, thus appropriate in the subsistence farming context. *Bemisia tabaci* is parasitized by an estimated 34 species of *Encarsia*, 14 species of *Eretmocerus*, and several species belonging to the genera *Amitus* and *Metaphycus* worldwide [21]. The parasitic wasps *Encarsia* spp. and *Eretmocerus* spp. are known to utilize the cassava whitefly *B. tabaci* as their host in East Africa [20,22,23,24], in Cameroon [25], and in Burkina Faso [26]. Six species of *Encarsia* were reported in Tanzania, with *Encarsia sophia* Dodd and Girault as the most prevalent parasitoid, followed by *Encarsia lutea* Masi. *Eretmocerus* has four species, with *Eretmocerus mundus* Mercet as the most prevalent [20]. The intensive utilization of insecticides for the control of arthropod pests is a significant factor in the disruption of biological control by parasitoids and predators through reduction in population density and activity in the field [27,28]. Options are employed that minimize the impact of insecticides on biological control agents, such as reduced application, the use of selective and less persistent chemistries, targeted application, and changes in mode of application [27,28].

The electrical penetration graph (EPG) technique is a powerful tool used to study the feeding behavior of piercing-sucking insects, including whiteflies. EPG creates an electric circuit through the insect and the plant and measures the fluctuations in voltage in real-time while the insect is feeding, producing the waveforms that describe the feeding behaviour in detail [29]. The EPG technique has been widely used on *B. tabaci* in studies focusing on virus transmission, host resistance factors, and insecticide effects [30,31,32,33,34,35].

Cassava cuttings as seed material permit insecticide application with the soaking of the cuttings. This provides a protection for the initial growth phase. Research plot testing of flupyradifurone through cassava cutting dipping showed that it is effective at reducing adult whiteflies in the range of 46–83% and nymphs by 64–91% in small research plots [36]. The current study aimed to test the efficacy of cutting dipping in a real farming situation through farmer participatory trials and to assess the impact of flupyradifurone on whitefly parasitism. In addition, we ran laboratory experiments to monitor the probing behaviour of cassava whitefly on plants established from cuttings dipped in flupyradifurone versus non-treated control plants using the electrical penetration graph (EPG) technique. EPG elucidates the impact of systemic flupyradifurone one month after cutting dipping, giving us a detailed understanding of the whitefly probing and feeding time, at the nexus of the insecticide uptake, as well as the virus transmission. Other EPG studies have so-far focused on direct foliar spray application, monitored within days after application [33,37,38,39].

## 2. Materials and Methods

### 2.1. Whitefly Abundance and Virus Incidence—Farmer Field Study

#### 2.1.1. Plant Materials, Insecticide, Farmer Selection, and Field Planting

Ten volunteer farmers were recruited through collaboration with agricultural extension officers within the selected village named Mkuchembe (7°21′18″ S 39°05′51″ E · 92.2 m) in the Mkuranga District of the Pwani Region, Tanzania. The location selected has tropical weather conditions with a bi-modal rainfall pattern that comprises a long rainfall season (Masika) between March and June and a short rainfall season (Vuli) between October and December [40]. This location was selected because farmers encounter high incidences of CMD and CBSD and abundant populations of the whitefly vector *B. tabaci*. The trials were established during the long rainfall season of Masika (planted 3 March 2021). Each farmer agreed to prepare a plot of 50 m × 20 m size. The farms were randomly selected with a separation distance of 1 to 1.5 km apart. Cassava virus-free planting materials of the variety Kiroba were bought from a local authorized cassava stem producer in Kimanzichana, Mkuranga, for all 10 farmers. The insecticide flupyradifurone (Sivanto^®^ 200SL) was provided by Bayer AG (Monheim, Germany).

The cassava stems were cut into 15 cm long cuttings, each with 5 to 7 nodes. Each of 10 farms was divided into two sections, such that there were 20 × 20 m arrays of 400 untreated cassava cuttings that served as the control and 20 × 20 m arrays of 400 cassava cuttings that were treated with flupyradifurone. For treating with flupyradifurone, the 400 cassava cuttings were divided into two groups of 200 cassava cuttings, and each group was tied together and dipped for 1 h in 160 L diluted insecticide solutions contained in plastic drums. The dividing was performed purposely to allow the tied stack to fit in the drums properly. The cuttings both for control and treated were planted in respective farm portions at a spacing of 1 m × 1 m within and between rows. The manufacturer-recommended concentrations were used for flupyradifurone at 100 g a.i/100 L. The cuttings in the control were not dipped in water; this was necessary to demonstrate the practical application of this technology under farmer field conditions.

#### 2.1.2. Data Collection on Whitefly Abundance, Cassava Virus Incidence, and Root Yield

The percentage of sprouted cuttings was recorded as the proportion of sprouted cuttings out of the 400 planted each for flupyradifurone and non-treated control 3 weeks after planting. A central square plot with 100 plants was then selected, and 30 plants were randomly picked and tagged for data collection. Assessment was performed monthly for a period of six months for whitefly and nymph counts, as well as for the incidence and severity of CMD and CBSD. Parasitism was evaluated on 4-month-old plants (4 months after planting). Adult whiteflies were counted on the top five fully opened leaves, while whitefly nymphs were counted on the 9th and 10th leaves from the top of the plant (lowest two fully opened leaves in the case that plants did not have 10 leaves). CMD and CBSD were assessed visually on the same plants, and foliar symptom severity was recorded using the following method. CMD severity was assessed using the 1–5 scoring scale, where 1 = cassava plant showing no leaf symptoms; 2 = mild distortion and mild chlorosis on the leaves; 3 = significant distortion and chlorosis on one-third of most leaves; 4 = extreme distortion and presence of mosaic patterns on two-third of most leaves and general reduction of leaf size; and 5 = very severe mosaic symptoms on all leaves, appearance of distortion, twisting, misshapen, and severe leaf reduction of most leaves, accompanied by the severe stunting of plants [41]. CBSD plants were assessed using a score of 1–5, where 1 = no apparent symptoms; 2 = slight foliar feathery chlorosis and no stem lesions; 3 = prominent foliar feathery chlorosis, mild stem lesions, and no dieback; 4 = severe foliar feathery chlorosis, severe stem lesions, and no dieback; and 5 = defoliation, severe stem lesions, and dieback [42]. CMD and CBSD incidences were recorded as the proportion of plants with symptoms out of the 30 plants that were tagged for data collection. In addition, three plants were randomly selected from each farm to record stem height and number of stems per cutting planted. At 10 months after planting (MAP), the 30 plants from which monthly data were collected were harvested, and the yield in grams and number of roots per plant were determined. The CBSD root severity was assessed on all harvested roots from each plant using the 1–5 scoring scale, where 1 = no apparent necrosis, 2 = ≤ 5% of root necrosis, 3 = 6–10% of root necrosis, 4 = 11–25% of root necrosis and mild root constrictions, and 5 ≥ 25% of root necrosis with severe root constriction [42]. The cassava whitefly haplogroup that colonizes cassava in the Mkuranga District of the Pwani Region, Tanzania, where this study was carried out, is predominantly sub-Saharan Africa East and Southern Africa (SSA-ESA—mitotype SSA1-SG3). The other haplogroups have so far not been detected in this region [10].

### 2.2. Examination of Sampled Leaves for Whitefly Nymphs and Parasitism Rate

The plants used in this activity were selected from the ten farms that had flupyradifurone-treated and non-treated control plots, and using the same plots that were used to monitor whitefly abundance, CMD and CBSD. Leaves were stripped off four randomly selected 4-month-old plants (4 months after planting) per treatment, and leaves were arranged to maintain their order. In total, eight plants were selected per field, totalling 80 plants from all ten fields and equating to 40 control plants and 40 flupyradifurone-treated plants. All leaves from the 80 plants were harvested in a single day. The ordered leaves from young to old (top to bottom in cassava) were stuck in between two long strips of masking tape with spacing in between and placed in fabric bags to prevent condensation and rotting. Samples were transported in a cool box and stored at 9 °C in a cool room over the counting period.

The undersides of leaves were inspected through a stereo microscope. The 2nd, 3rd, and 4th instar nymphs and pupa stages were identified and recorded based on size and shape. The condition of the instar stages was differentiated by checking for green-paired mycetomes indicating a healthy instar; glossy flattened instar shell with no mycetomes indicating predation; and pale instars with orange discolored mycetomes, indicating death of unknown cause. *B. tabaci* pupae and nymphs parasitized by *Eretmocerus* spp., *Encarsia sophia*, and *En. lutea* were identified using standard morphological techniques [22].

### 2.3. Probing Behaviour of Whiteflies on Flupyradifurone-Treated Cassava Plants

#### 2.3.1. Whitefly Colony and Establishment of Flupyradifurone-Treated Plants

The *B. tabaci* haplogroup used in this study was from sub-Saharan Africa East and Southern Africa (SSA-ESA—mitotype SSA1-SG3) [10]. The whiteflies were collected from cassava plants in Chambezi in the Bagamoyo District, Coast Region, Tanzania, in May 2019 and introduced to potted cassava plants placed in 50 × 50 × 100 cm netted cages. The cassava plants were grown in 7.5 L pots containing a mixture of soil and farmyard manure at a 4:1 ratio, and the whitefly colonies were reared on the cassava plants in the screen house at 25–30 °C and 65–75% RH. Whiteflies were transferred to fresh one-month-old cassava plants at intervals of four to six weeks to maintain the colonies. The cassava plants were established in a screenhouse at IITA-Tanzania, Dar es Salaam, using the whitefly-preferred cassava variety Albert. Cassava cuttings were dipped into solutions of flupyradifurone (100 g a.i/100 L) or water for 1 h. A total of 40 cuttings were planted individually in 1 L pots containing a mixture of soil and farmyard manure for each treatment. Thirty-two plants were selected and used for EPG experiments at the four-leaf stage.

#### 2.3.2. Electrical Penetration Graph (EPG) Technique

The probing and feeding behaviours of whiteflies on plants treated with flupyradifurone vs. the control were monitored using the electrical penetration graph (EPG) technique [29]. The experiments were carried out using the Giga-8d DC-EPG device, which has an input resistance of 1 Giga-ohm and can record up to 8 insects at once (EPG systems, Wageningen, The Netherlands). A 1 cm long, 2.5 μm thick platinum wire (Sigmund Cohn Corp., Mt Vernon, NY, USA) was attached to the top of the head of the test insect (adult female whitefly) using electrically conductive silver glue (EPG Systems, Wageningen, The Netherlands). The other end of the platinum wire was attached to a 2.5 cm copper wire, which was then soldered to a brass nail inserted into the EPG probe. The platinum wire was thin and flexible, which allowed the attached insect to move around freely [35]. One insect was placed on one plant, and plants were used only once. Insects were placed on the abaxial surface of the second leaf from the top of the plant. To access the lower surface, leaves were inverted, and their bases were taped to a solid surface with electrical tape. The abaxial surface was used since it is usually the preferred feeding site for whiteflies. Eight plants were recorded at one time for 12 h. Four of the eight were flupyradifurone-treated, and the other four were water-treated controls. A total of 32 plants were recorded per treatment for flupyradifurone and the control. The EPG waveforms selected for analysis included the following distinct waveforms: (i) C: the intercellular apoplastic stylet pathway where the insects showed the cyclic activity of mechanical stylet penetration and secretion of saliva; (ii) Pd: potential drops resulting from intracellular stylet puncture occurring during the stylet pathway; (iii) E1: salivation into phloem sieve elements at the beginning of the phloem phase; (iv) E2: passive phloem sap uptake from the sieve element; (v) G: the active intake of xylem sap; (vi) Np: non-probing, where insect stylets are withdrawn from the leaf [35].

### 2.4. Data Analysis

The field experiment data were subjected to a one-way analysis of variance (ANOVA) using PROC GLIMMIX (SAS version 9.4; SAS Institute, Cary, NC, USA) with Poisson distribution for whitefly adults, nymph numbers, and parasitoid numbers and lognormal distribution for percent cutting sprouting, percent incidence of CMD and CBSD, percent parasitism, and yield data. The fixed factor was treatment, and the random factors were farmers’ plots and replications. The flupyradifurone treatment was considered significant at *p* = 0.05. The LSMEANS statement was used to obtain least squares means, and the Tukey-Kramer test was used for means separation. Treatment means and standard errors were obtained using the PROC MEANS statement in SAS. The disease severity scores for CMD and CBSD were subjected to a non-parametric Kruskal–Wallis test. Raw EPG data were recorded by EPG Systems Stylet+d and manually annotated using EPG Systems Stylet+a software v01.30. The annotated files were imported into the EPG_analysisworksheet_v4.4.3.xls, which contained a worksheet and data subjected to analysis of variance using PROC GLIMMIX with lognormal. The flupyradifurone treatment was considered significant at *p* = 0.05. The LSMEANS statement was used to obtain least squares means, and the Dunnett test was used for the comparison of treatment to the control. Treatment means and standard errors were obtained using the PROC MEANS statement in SAS.

## 3. Results

### 3.1. Whitefly Abundance, Cassava Mosaic, and Brown Streak Disease Incidence and Root Yield

**Figure 1:**
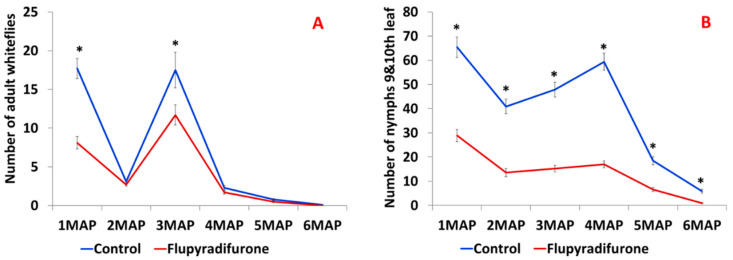
Adult whitefly numbers recorded from the top five leaves for a six-month period (**A**), and nymphs on the 9th and 10th leaves (**B**). Means denoted with an asterisk (*) are significantly different (*p* = 0.05, Tukey-Kramer test). MAP = months after planting.

The adult whitefly numbers were in the range of 0–18 in the control group and 0–12 in the flupyradifurone group. The differences were only significant (*p* < 0.05) at 1 month after planting (MAP), 3MAP, and 5MAP, and on average, there was a 41% reduction in numbers on treated plants (Figure 1A). The number of nymphs ranged from 6 to 65 for the non-treated control and 1 to 29 for flupyradifurone-treated plants. On average, there was a 65% reduction in nymphs on treated plants. This significant reduction (*p* < 0.0001) persisted over the entire six-month period (Figure 1B).

**Figure 2:**
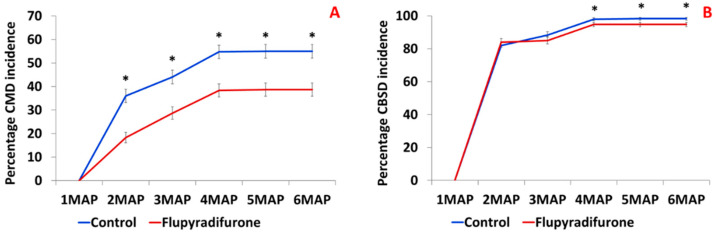
The percentage incidence of cassava mosaic disease (CMD), (**A**) and cassava brown streak disease (CBSD) (**B**) recorded for a six-month period. Means denoted with an asterisk (*) are significantly different (*p* = 0.05, Tukey-Kramer test). MAP = months after planting.

The incidence of CMD was significantly lower (*p* < 0.0001) in flupyradifurone compared to the control for the six-month duration (Figure 2A). The incidence range for non-treated control plants was 0–55% with an average of 41%, while the treated plants had a range of 0–39% with an average range of 27%. The CBSD incidence was not significantly different in the control group and flupyradifurone group (*p* > 0.05). The incidence was 0% at 1MAP after planting, but in subsequent months, it was in the range of 82–98% with an average of 76% in both treated and non-treated control plants (Figure 2B). The disease severity of CMD was significantly lower (*p* < 0.05) in flupyradifurone-treated plants compared to control plants at 3MAP, 4MAP, 5MAP, and 6MAP (Table 1). The severity of CBSD foliar symptoms was significantly lower in flupyradifurone-treated compared to control plants at 5MAP and 6MAP (Table 1).

The cutting sprouting in plants treated with flupyradifurone was not significantly different (*p* = 0.6046) compared to control plants (Table 2). The plants treated with flupyradifurone produced significantly longer stems (*p* < 0.0001) by 46% compared to non-treated control plants at 6MAP (Table 2). The average number of stems per cutting at 6MAP was slightly higher in the flupyradifurone treatment, but this difference was not significant compared to non-treated control plants (Table 2). The number of roots in plants treated with flupyradifurone was significantly greater (*p* < 0.0001) by 38%, while the root weight was significantly higher (*p* < 0.0001) (49%) compared to non-treated control plants (Table 2). The incidence of CBSD in roots was very low (<1%) with only 1 out of 300 plants in the control group and 3 out 300 plants in the flupyradifurone group showing mild necrosis (score 2 ≤ 5% of root necrosis).

### 3.2. Whitefly Nymphs and Parasitism Rate

Whitefly stages and parasitized pupae were counted from leaves stripped from entire selected plants per each treatment. The mean total number of eggs, first stage, and fourth stage nymphs were significantly lower (*p* < 0.0144) on flupyradifurone-treated plants compared to non-treated control plants, but there were no significant differences for second and third stage nymphs (Table 3). The mean total number of parasitoids *Eretmocerus* spp. was significantly less (*p* = 0.0216) on flupyradifurone-treated plants compared to non-treated control plants, while the numbers for *Encarsia sophia* and *E. lutea* were not significantly different (Table 3). The percentage of parasitism calculated as the proportion of parasitized nymphs to total nymph counts (sum of parasitized and unparasitized nymphs) showed there were no significant differences for *Encarsia* spp. on flupyradifurone-treated plants compared to non-treated control plants for individual parasitoid species. Combined parasitism (*Encarsia* spp. and *Eretmocerus* spp.) showed that the flupyradifurone treatment was marginally lower but not significantly different (*p* = 0.0551) compared to the control plots (Table 3).

### 3.3. Probing Behaviour of Whiteflies on Flupyradifurone Treated Cassava Plants

A total of 18 whitefly EPG probing behaviours were reported in this study (Table 4). The time from the start of the EPG to the first phloem phase (E) and the time from the start of probing to the first E were significantly shorter (*p* < 0.05) by 1.9 times in non-treated control plants compared to flupyradifurone-treated plants. The total probing duration (C) was significantly longer (*p* = 0.0125) in control plants compared to flupyradifurone-treated plants, while the total non-probing (np) duration was significantly longer (*p* = 0.0105) by two times in treated plants compared to control plants. The mean np duration in flupyradifurone-treated plants was significantly longer (*p* = 0.0005) by five times compared to the control. The total potential drop duration (pd) was significantly longer (*p* = 0.0399) by two times on control plants compared to flupyradifurone-treated plants. The phloem ingestion total and mean durations were 1.8 and 2 times longer, respectively, in control compared to flupyradifurone-treated plants, although these differences were not significant (Table 4).

## 4. Discussion

The whitefly *Bemisia tabaci* is a damaging pest of numerous crops worldwide. This study sought to evaluate the efficacy of cassava cutting dipping in flupyradifurone against cassava whiteflies under farmer field participatory trials and to assess the impact on whitefly parasitism. The cutting dipping method for flupyradifurone proved effective against cassava whitefly *B. tabaci* haplogroup SSA-ESA (mitotype SSA1-SG3) under screen house and small research plot experiments [36].

The farmer participatory study reported here was carried out during the long rainfall (Masika) season because farmers prefer planting cassava during this season, although whiteflies are known to be abundant during the short rainfall (Vuli) season [36,43]. Surprisingly, the farmer fields had abundant whiteflies that were comparable in numbers to those usually recorded during the Vuli season, and the nymphs at 6MAP in control plots were in the range of 5–65 compared to 7–43 that was reported for a Vuli season experiment [36]. This could be attributed to the unusually dry conditions experienced during the study period.

The findings from this experiment showed that dipping cassava cuttings in flupyradifurone before planting is effective at reducing whitefly numbers and nymphs for up to a duration of six months under farmer field conditions. The incidence and severity of CMD was also significantly reduced, a fact that is a likely consequence of the reduced whitefly populations in treated plants. Previous studies under controlled conditions showed that the application of flupyradifurone reduced the virus transmission of Tomato yellow leaf curl virus [16,44] and Cucurbit yellow stunting disorder virus [45]. Tomato yellow leaf curl virus incidence was significantly reduced on tomatoes under field conditions in flupyradifurone-treated plots compared to the control [46]. The incidence of CBSD was high from an early stage, reaching an average of 83% at 2MAP, but the severity score was in the range of 2.46 to 2.96 which corresponded to foliar feathery symptoms. This suggests that there was a very high inoculum pressure from surrounding infected fields. The viruses that cause CBSD are transmitted semi-persistently by *B. tabaci* [47], which means that plants can be rapidly inoculated but that the virus is not retained for long periods, meaning that most transmission occurs over short distances [48]. The mild severity of foliar symptoms of CBSD throughout the six-month duration, despite the early infection of plants, and the <1% root infection incidence indicate that the variety Kiroba has some tolerance to CBSD [43]. By contrast, the begomoviruses that cause CMD are persistently transmitted, which means that inoculation takes several hours of feeding, but viruses can be carried throughout the insects lifetime and spread over relatively long distances [49]. These facts mean that CMD is more readily controlled using insecticides, as feeding adults are likely to die before inoculation has occurred. It should also be noted that having an untreated control adjacent to the treated plots meant that the effectiveness of the insecticidal treatment was compromised to some degree. This could be addressed in the future by establishing treated and control plots in different locations. For farmers applying cutting dip treatments, however, it is anticipated that the benefits of virus control would be greater than those demonstrated in this experiment, as they would treat their entire field rather than just a portion.

The yield increase of 49% shows that farmers who adopted the use of flupyradifurone-treated cuttings, based on yield obtained in this study, could harvest approximately 11.5 t/Ha compared to non-treated cuttings with a yield of 7.7 t/Ha. This significant increase in yield shows that whitefly damage alone in cassava, apart from the transmission of viruses, also does cause high yield losses. The stems in treated fields were on average 46% longer, which is an indication that farmers who sell stems could benefit more with treated cuttings. A previous study on cassava testing combined cutting dipping and spraying of imadicloprid and reported reduced whitefly numbers and CMD and CBSD incidence and a yield gain of approximately 50% in treated compared to control plots [50].

The use of chemical pesticides is one of the components deployed in IPM programs; therefore, it is critical to understand the effects of insecticides on parasitoid natural enemies, as this knowledge will help enhance the combined use of these control strategies [51,52,53,54]. Studies involving testing the effect of various insecticides on parasitoids revealed that toxicity does vary depending on insecticide chemistry and the timing of field applications in relation to the prevalence of natural enemies [52,55]. The presence of *Encarsia* spp. and *Eretmocerus* spp. parasitizing cassava *B. tabaci* in Tanzania is consistent with earlier findings that these are common species in East Africa [20,22,23]. The percent parasitism of these two species, 27.3% (*Encarsia* spp.) and 26.7% (*Eretmocerus* spp.), are equal in proportion to those reported previously, although earlier studies recorded *Encarsia* spp. as the predominant genus [22]. In Burkina Faso, *Eretmocerus* spp. was reported as the most predominant compared to *Encarsia* spp. [26]. Under natural field conditions, parasitoids have a limited impact on controlling cassava whitefly populations and the prevention of cassava virus transmission where vector populations are high [20,22]. The total parasitism percentages of 39% (flupyradifurone) and 54% (control) are comparable to the 40–58% earlier reported from farmer field surveys, the 26–42% under large-scale field experiments in Uganda [22,24], and the combined parasitism of 41.8% for *En. lutea* and *En. sophia* in Cameroon [25]. The level recorded here was higher than in Burkina Faso, where there was less than 20% parasitism of whiteflies in cassava [26]. The slight lower combined parasitism in flupyradifurone could be attributed to the lower availability of prey compared to the non-treated control plots, considering the latter had two times the number of fourth instar nymphs. Studies have reported a positive correlation between whitefly nymph abundance and parasitism rate under field conditions [25]. A previous field study in cotton revealed that reduced predation could be due to reduced prey in plots treated with pyrifluquinazon as opposed to toxicity to predators [56]. Flupyradifurone was also tested and found to have no significant effect on predators [56]. In a laboratory study comparing the effect of insecticides on the parasitoid *Trichogramma evanescens* Westwood through the dipping of parasitized eggs, flupyradifurone was categorised as the least harmful compared to spirotetramat and deltamethrin [57].

The cutting dipping application of flupyradifurone significantly reduced whitefly numbers and had minimal impact on individual parasitoid species. Imidacloprid has been examined for a few species of *B. tabaci* parasitoids, and results suggest that, while systemic applications are generally harmless, foliar applications can be highly toxic [27]. Non-selective systemic insecticides that are harmful to parasitoids and predators when applied through spraying may be altered to be selective by changing the mode of systemic delivery, for example by seed treatment through coating, cutting dips, and root dips for seedlings [28]. There is a need to adopt agricultural management practices that benefit natural enemies, such as the elimination of frequent spray applications of broad-spectrum insecticides [58]. Several studies have shown that parasitoid abundance or whitefly nymph parasitism tend to be lower in conventional farms that employ insecticidal sprays when compared to farms that utilize organic practices [58,59,60]. This is the first study to explore the effect of insecticide application through cutting dipping on whitefly parasitism in cassava farmer fields.

The time from the start of EPG to the first probe and the duration of the first probe were not significantly different between treated and controls, which is an indication that flupyradifurone was not a deterrent to the initial probing. A previous study reported similar findings, in which tomato plants sprayed with flupyradifurone 24 h before EPG recording were not different from the controls [37]. Flupyradifurone significantly reduced the probing time, which also coincided with an extended non-probing duration. These are indications that this treatment made the plant unfavourable for whitefly feeding and inhibited pathway activities compared to the control. Similar findings were reported in other studies, in which insecticides reduced whitefly pathway activities associated with feeding and virus transmission, even though most studies used plants treated within 24 h or a few days [33,38], as opposed to our study, in which plants were used almost at four weeks after cutting dipping insecticide application. Studies evaluating the effect of various insecticides on the feeding behaviour of *B tabaci* (acetamiprid, bifenthrin, cyantraniliprole, flupyradifurone, imidacloprid, and pymetrozine) reported reduced probing and phloem activity in treated plants compared to control non-treated plants [33,45,46,61,62]. Systemic insecticides are important for the control of semi-persistently and persistently transmitted viruses, as they reduce vector numbers and alter the feeding behaviours associated with virus transmission [45,46,63,64].

The cutting dip technology should be considered for incorporation in an IPM package for the control of whiteflies in cassava. This will be beneficial to non-target organisms and the environment as opposed to the use of the spray application method. The effectiveness of flupyradifurone in reducing whitefly populations up to six months after planting could play a critical role in reducing damage caused by this pest and the viruses it transmits. It is noted that the infection of cassava plants with CMD five months after planting does not significantly impact yield, as the plants would have already initiated root formation [65]. The first six months of cassava growth also coincide with those through which *B. tabaci* populations peak. Thereafter, they reduce drastically as plants grow taller, become woody and shade lower leaves [66]. The set of results presented here suggests that cutting dips will help cassava growers throughout sub-Saharan Africa to increase their yields, as well as produce increased quantities of planting material. As commercial opportunities for cassava seed and root production continue to increase across Africa, this component of improved cassava crop management could contribute significantly to strengthening this important part of the agricultural economy.

## 5. Conclusions

This study shows that the application of flupyradifurone in cassava through cutting dipping under farmer field conditions is effective at reducing cassava *Bemisia tabaci* whiteflies, which resulted in a 49% yield increase that could be attributed to reduced whitefly damage and CMD incidence. Flupyradifurone application through cutting dipping had no significant effect on parasitism by the individual species of *Encarsia* spp. and *Eretmocerus* spp. parasitoids, an indication that this mode of application would minimize effects on non-target organisms that are usually harmed through spray applications. This technology could be adopted as a component of IPM for the management of whiteflies in cassava and can be expanded to test other vegetatively propagated crops that are affected by whiteflies.

## Figures and Tables

**Table 1:** The disease severity scores of cassava mosaic disease (CMD) and cassava brown streak disease (CBSD) in cassava plants established from cuttings dipped in flupyradifurone in a farmer participatory field trial (Means) ^Z^.

Disease	MAP	Flupyradifurone	Control	*χ*^2^ Value	*p* Value
CMD	1MAP	-	-	-	-
	2MAP	2.96	3.14	2.22	0.1367
	3MAP	2.80	3.11	14.84	0.0001
	4MAP	2.92	3.10	7.23	0.0071
	5MAP	2.92	3.10	7.25	0.0071
	6MAP	2.91	3.12	10.00	0.0016
CBSD	1MAP	-	-	-	-
	2MAP	2.65	2.69	1.04	0.3071
	3MAP	2.50	2.46	0.98	0.3222
	4MAP	2.71	2.74	0.48	0.4889
	5MAP	2.85	2.93	8.41	0.0037
	6MAP	2.89	2.96	10.94	0.0009

^Z^ Means with corresponding *p* > 0.05 within rows are not significantly different (Kruskal–Wallis test).

**Table 2:** The yield parameters of cassava plants established from cuttings dipped in flupyradifurone in a farmer participatory trial (Means ± SE) ^Z^.

Parameter	Flupyradifurone	Control	*p* Value
Cutting sprouting %	90.1 ± 3.0 a	87.8 ± 3.1 a	0.6046
Stem height in cm	124.3 ± 6.6 b	84.6 ± 5.0 a	<0.0001
Number of stems/plant	2.9 ± 0.3 a	2.4 ± 0.2 a	0.1170
Number of roots/plant	4.7 ± 0.2 b	3.4 ± 0.1 a	<0.0001
Root yield in g/plant	1150.6 ± 42.5 b	769.5 ± 34.1 a	<0.0001

^Z^ Means with the same letter within rows are not significantly different (*p* = 0.05, Tukey-Kramer test).

**Table 3:** Mean total counts of *Bemisia tabaci* life stages and parasitized pupa and percent parasitism on 4-month-old cassava plants established from cuttings treated with flupyradifurone (Means ± SE) ^Z^.

Whitefly Stage	Control	Flupyradifurone	*p* Value
Egg	1510.8 ± 374.4 b	686.5 ± 154.9 a	0.0144
1st stage nymphs	533.9 ± 111.1 b	278.6 ± 65.9 a	0.0490
2nd stage nymphs	44.4 ± 7.0 a	37.1 ± 11.4 a	0.5412
3rd stage nymphs	39.0 ± 7.5 a	39.2 ± 8.1 a	0.9813
4th stage nymphs	41.2 ± 8.8 b	20.8 ± 3.4 a	0.0260
Pupa	24.2 ± 4.5a	16.0 ± 3.4 a	0.1586
Parasitoids			
*Encarsia sophia*	14.9 ± 4.4 a	7.6 ± 2.1 a	0.1544
*Encarsia lutea*	2.3 ± 1.4 a	1.6 ± 1.2 a	0.6154
*Eretmocerus* spp.	17.0 ± 3.6 b	6.4 ± 2.0 a	0.0216
Percent parasitism			
*Encarsia sophia*	23.5 ± 4.4 a	18.1 ± 3.7 a	0.9931
*Encarsia lutea*	3.8 ± 1.7 a	3.1 ± 1.9 a	0.4952
*Eretmocerus* spp.	26.7 ± 4.1 a	18.0 ± 3.6 a	0.4119
Total parasitism	54.0 ± 4.8 a	39.2 ± 5.2 a	0.0552

^Z^ Means with the same letter within rows are not significantly different (*p* = 0.05, Tukey-Kramer test).

**Table 4:** EPG parameters of *Bemisia tabaci* on cassava plants generated from cuttings that were dipped in flupyradifurone (Means ± SE) ^Z^.

Parameter	Control	Flupyradifurone	*p* Value
Time to 1st probe from start of EPG	3.5 ± 1.6 a	8.9 ± 2.8 a	0.4026
Duration to 1st probe	54.6 ± 32.7 a	10.9 ± 3.2	0.5899
Time from start of EPG to 1st E	262.4 ± 42.6 a	495.2 ± 47.7 b	0.0103
Time from 1st probe to 1st E	258.8 ± 43.3 a	486.3 ± 48.0 b	0.0205
Total duration of G	59.5 ± 10.7 a	92.1 ± 27.2 a	0.9294
Total duration of E1	6.3 ± 3.2 a	1.5 ± 0.3 a	0.4077
Total duration of E2	283.1 ± 43.7 a	158.4 ± 59.1 a	0.0759
Total duration of C	183.8 ± 19.5 b	119.7 ± 16.8 a	0.0124
Total duration of np	160.1 ± 38.2 a	350.9 ± 40.0 b	0.0105
Total duration of pd *	189.7 ± 35.1 b	99.6 ± 26.4 a	0.0399
Total duration of F	53.3 ± 6.5 a	104.8 ± 19.9 a	0.6563
Mean duration of G	31.0 ± 7.8 a	37.3 ± 11.8 a	0.7387
Mean duration of E1 *	83.3 ± 31.6 a	55.9 ± 10.6 a	0.6442
Mean duration of E2	141.2 ± 33.3 a	71.7 ± 28.8 a	0.1444
Mean duration of C	9.9 ± 0.9 a	8.7 ± 1.1 a	0.4524
Mean duration of np	8.3 ± 2.1 a	42.0 ± 11.5 b	0.0005
Mean duration of pd *	6.3 ± 0.4 a	5.9 ± 0.5 a	0.5011
Mean duration of F	19.9 ± 1.9 a	30.3 ± 5.5 a	0.7135

^Z^ Means with the same letter within rows for control vs. flupyradifurone are not significantly different (*p* = 0.05, Dunnett test). C, stylet pathway and sheath salivation; G, xylem ingestion; E1, phloem salivation; E2, phloem ingestion; pd, potential drops; np, non-probing; F, difficulties in the stylet during probing and unknown waveforms; * duration in seconds.

## Data Availability

Data for this study will be available upon request.
